# ^18^F-FDG and ^68^Ga-somatostatin analogs PET/CT in patients with Merkel cell carcinoma: a comparison study

**DOI:** 10.1186/s13550-018-0423-3

**Published:** 2018-07-21

**Authors:** Silvia Taralli, Martina Sollini, Michele Milella, Germano Perotti, Angelina Filice, Massimo Menga, Annibale Versari, Vittoria Rufini

**Affiliations:** 1 Department of Diagnostic Imaging, Radiation Oncology and Hematology, Nuclear Medicine Unit, Fondazione Policlinico Universitario A. Gemelli IRCCS, Roma, Italia; 2 Department of Biomedical Sciences, Humanitas University, Pieve Emanuele (Milan), Italy; 3 Division of Medical Oncology, Regina Elena National Cancer Institute, Rome, Italy; 4 Nuclear Medicine Unit, Arcispedale Santa Maria Nuova—IRCCS Reggio Emilia, Reggio Emilia, Italy; 5 Department of Diagnostic Imaging, Radiation Oncology and Hematology, Institute of Nuclear Medicine, Fondazione Policlinico Universitario A. Gemelli IRCCS, Roma—Università Cattolica del Sacro Cuore, Largo A. Gemelli, 8, 00168 Roma, Italia

**Keywords:** Merkel cell carcinoma, Positron emission tomography/computed tomography, ^18^F-FDG, ^68^Ga-somatostatin analogs

## Abstract

**Background:**

Merkel cell carcinoma (MCC) is an aggressive neuroendocrine skin tumor. Currently, ^18^F-fluoro-deoxy-glucose (^18^F-FDG) PET/CT is the functional imaging modality of choice. Few data are available on the use of ^68^Ga-somatostatin analogs. The aim of our study was to evaluate and compare the diagnostic performance of ^18^F-FDG and ^68^Ga-somatostatin analog PET/CT in MCC patients.

**Results:**

Fifteen patients (12 males, 3 females; median age 73 years; range 41–81 years) with histologically proven MCC (4 with unknown primary lesion) who underwent both ^18^F-FDG and ^68^Ga-somatostatin analog PET/CT for staging, re-staging, or treatment response assessment were retrospectively evaluated. Results of both studies were qualitatively analyzed and compared on a patient- and lesion-based analysis, using histology or clinical/radiological follow-up as reference standard for final diagnosis. According to final diagnosis, 8/15 patients had at least one MCC lesion and 7/15 had no evidence of disease. On a patient-based analysis, ^18^F-FDG and ^68^Ga-somatostatin analogs correctly classified as positive 8/8 (100% sensitivity) patients and as negative 6/7 (85.7% specificity) and 5/7 (71.4% specificity) patients, respectively, with no significant difference. On a lesion-based analysis, ^18^F-FDG detected 67/75 lesions (89%) and ^68^Ga-somatostatin analogs 69/75 (92%), with no significant difference. In four patients with unknown primary MCC, both tracers failed to identify the primary MCC site.

**Conclusions:**

Our preliminary data suggest that ^18^F-FDG and ^68^Ga-somatostatin analog PET/CT provide good and equivalent diagnostic performance, adding interesting insights into the complex MCC biology. However, these results do not suggest that ^18^F-FDG PET/CT should be replaced by ^68^Ga-somatostatin receptor imaging, which should be performed in addition, according to clinical indication, to the perspective of “personalized medicine.”

## Background

Merkel cell carcinoma (MCC) is a rare highly aggressive skin tumor, with a quickly increasing incidence rate over the last 20 years. It is characterized by neuroendocrine features such as somatostatin receptor expression, besides a frequently high mitotic index, tumor necrosis, and vascular invasion [1–3]. MCC shows rapid local growth, high incidence of both regional lymph node and distant metastases, high rate of local recurrence, and a poor prognosis [1–4]. The primary tumor site cannot be found in up to 25% of patients (MCC of unknown primary origin; UPMCC) [5].

Currently, the optimal imaging algorithm of MCC is not yet defined [6, 7]. ^18^F-fluoro-deoxy-glucose (^18^F-FDG) PET is a valuable functional modality to image MCC, due to the increased glucose metabolism which reflects its clinical aggressiveness [8–15]. According to the European consensus-based interdisciplinary guideline, ^18^F-FDG PET/CT or CT scan should be performed as part of the initial work up of MCC to stage the disease [16], while according to the more recent National Comprehensive Cancer Network (NCCN) guidelines, ^18^F-FDG PET/CT may be preferred to CT [17]. Moreover, ^18^F-FDG PET/CT is suggested as a routine imaging study during follow-up, as clinically indicated [16, 17]. Due to the neuroendocrine aspects of MCC cells, somatostatin receptor scintigraphy (SRS) with ^111^In-pentetreotide (Octreoscan®) has been initially used with variable results [18–20]. Recent evidences regarding the use of somatostatin analogs labeled with positron emitters (^68^Ga-peptides) for PET imaging in MCC have shown positive results, suggesting a possible impact on MCC management [21–24]. A direct comparison between ^18^F-FDG and ^68^Ga-somatostatin receptor imaging (^68^Ga-SRI) PET/CT in the same MCC patient has been only reported in a few case reports [25–27]. In this regard, potential interesting insights into tumor biology could derive from a head-to-head comparison of both tracers, besides possible implications also in the perspective of a personalized patient management.

The aim of our study was to evaluate and compare the diagnostic performance of ^18^F-FDG and ^68^Ga-SRI PET/CT in patients with MCC.

## Methods

### Patients

All consecutive patients with a previous histological diagnosis of MCC who underwent both ^18^F-FDG PET/CT and ^68^Ga-SRI PET/CT at the PET/CT center of “Fondazione Policlinico Universitario A. Gemelli IRCCS” in Roma (center A) or “Arcispedale Santa Maria Nuova-IRCCS” in Reggio Emilia (center B) between September 2007 and May 2014 were considered. Inclusion criteria for this retrospective study were as follows: availability of ^18^F-FDG and ^68^Ga-SRI PET/CT scans performed within a 6-week interval (considering the rapid progression of MCC), no interval therapeutic procedures performed between the two scans, and availability of histopathological proof of imaging findings or, alternatively, clinical-radiological data on follow-up as reference standard for the final diagnosis. Sixteen consecutive patients were considered; one patient was excluded because he underwent surgical excision of the primary MCC lesion in the interval period between the two PET/CT scans. Therefore, 15 patients (12 males and 3 females; median age 73 years, range 41–81 years) were finally analyzed: 8 from center A and 7 from center B. This cohort of patients was also included in a previous bi-center study evaluating the role of ^68^Ga-SRI PET/CT in 23 MCC patients [21]. According to standard clinical protocols in use in both institutions, this retrospective study was approved by the local ethics committees and an informed consent for both PET/CT examinations and the use of personal data was obtained from all patients.

### ^18^F-FDG PET/CT

^18^F-FDG PET/CT studies were performed according to a previously described protocol [28]. Briefly, all patients presented blood glucose levels under 8.3 mmol/L at the time of tracer injection, and they were in optimal hydration state. PET/CT images were acquired 60 ± 10 min after intravenous injection of a mean of 293 ± 72 MBq of ^18^F-FDG according to body weight.

### ^68^Ga-SRI PET/CT

Patients were imaged with different radiolabeled somatostatin analogs: ^68^Ga-DOTANOC was used in center A (eight patients), whereas ^68^Ga-DOTATOC or ^68^Ga-DOTATATE were used in center B (five and two patients, respectively). In center A, ^68^Ga was obtained from a ^68^Ge/^68^Ga generator (IGG 100; Eckert & Ziegler Isotope Products, Berlin, Germany), with a nominal activity of 1.85 GBq. DOTANOC was labeled with ^68^Ga as previously described [29]. In center B, ^68^Ga was obtained from a commercially available ^68^Ge/^68^Ga generator (Ciclotron, Napa, CA, USA) with a nominal activity of 1.85 GBq. DOTATOC and DOTATATE were labeled with ^68^Ga according to a previously described protocol [30]. The administered activity of ^68^Ga-somatostatin analogs was 2–2.5 MBq/kg. Images were acquired 60 ± 10 min after tracer injection, as previously described [21].

### Images interpretation and data analysis

All ^18^F-FDG and ^68^Ga-SRI PET/CT images were independently evaluated by two experienced nuclear medicine physicians (ST and MS), aware of the clinical and follow-up reports. Any disagreement was resolved by consensus. A qualitative image analysis was performed: all PET/CT studies were classified as positive if at least one abnormal area of focal tracer uptake outside the physiological distribution or higher than the surrounding physiological activity was detected [31, 32]. The number sites and maximum standardized uptake value (SUVmax) of all abnormal findings identified on both PET/CT scans were also recorded for each patient. The histopathological proof (as available) or, alternatively, a combination of morphological and functional studies and clinical information during follow-up were used as reference standard to verify PET/CT results. Accordingly, functional imaging findings were classified as true positive, false positive, true negative, or false negative for MCC-related lesions. The results of the two PET/CT studies were compared on a patient-based and on a lesion-based analysis.

A clinical decision was made considering all imaging modality results. The impact on patient’s management was assessed evaluating whether additional information provided by ^18^F-FDG PET/CT and/or ^68^Ga-SRI PET/CT had influenced the clinical decision in comparison to the expected patient management based on the results of the available pre-PET morphological imaging.

### Statistical analysis

A descriptive analysis was performed. For patient-based analysis, the diagnostic performances of ^18^F-FDG and ^68^Ga-SRI PET/CT in detecting MCC lesions were calculated in terms of sensitivity and specificity, and compared by the chi-square test with Yates’ correction. Results were reported with 95% confidence intervals (CI). For lesion-based analysis, the Wilcoxon signed rank test was used to compare differences in the number of true positive lesions detected by ^18^F-FDG and ^68^Ga-somatostatin analogs. A *p* value < 0.05 was considered significant. Data were analyzed by MedCalc Statistical Software version 15.11.4.

## Results

Detailed demographic and clinical features of the study population are listed in Table 1. Histological diagnosis of MCC was obtained by surgical removal of the primary lesion in 11/15 patients and by excisional biopsy of lymph node lesions in the remaining 4/15 patients with no identifiable primary tumor site (UPMCC). Clinical indications for PET/CT examination were staging at initial diagnosis (4/15), post-surgical staging (3/15), re-staging for suspected recurrence at imaging (3/15) or for clinical progression (2/15), and post-chemotherapy evaluation (3/15). In this comparative study, ^18^F-FDG PET/CT was the first imaging of choice for evaluating MCC, according to current international guidelines [17]. PET/CT results were confirmed by histology in one case (lymph node surgical excision) and by a combination of imaging data (diagnostic CT, MRI, ultrasound imaging, chest X-ray, further PET/CT scan) and clinical information during follow-up in the remaining 14 patients. The median follow-up time was 34 months (range 7–122 months). At the end of follow-up, 8/15 patients were dead (MCC-related death in 7 cases) and the remaining 7 patients were alive.

**Table 1:** Demographic and clinical characteristics of the study population (*n* = 15)

Patient	Gender, Age (years)	Site of MCC at diagnosis		MCC stage at diagnosis	Previous surgery for MCC	Previous treatment, time between previous treatment and PET/CT (months)	Associated malignancy	Clinical indication for PET/CT
		Primary MCC	Unknown primary MCC					
1	M, 74	Ear		III	Primary tumor excisional biopsy	–	Prostate cancer + chronic lymphocytic leukemia	Staging
2^*^	M, 72	Thigh		IV	Surgical excision + lymph node excisional biopsy	CHT (1)	–	Re-staging^#^
3	M, 74	Arm		III	Surgical excision + loco-regional lymphadenectomy	CHT (8)	–	Re-staging^§^
4	M, 80		Inguinal lymph node	III	Lymph node excisional biopsy	–	Colorectal cancer + cutaneous melanoma	Staging
5	F, 80	Cheek		III	Surgical excision + loco-regional lymphadenectomy	–	Chronic myeloid leukemia	Post-surgical evaluation
6	M, 73	Hand		III	Surgical excision + loco-regional lymphadenectomy	–	–	Re-staging^§^
7	M, 47	Cheek		II	Primary tumor excisional biopsy	–	–	Staging
8	M, 71		Inguinal lymph node	III	Lymph node excisional biopsy	CHT (1)	–	Post-CHT evaluation
9	M, 58	Thigh		III	Surgical excision + loco-regional lymphadenectomy	–	–	Post-surgical evaluation
10^*^	M, 79	Back		III	Surgical excision + loco-regional lymphadenectomy	CHT (4)	Pancreatic NET	Post-CHT evaluation
11^*^	M, 81	Thigh		III	Surgical excision + sentinel lymph node biopsy	RT (10)	Cutaneous melanoma	Re-staging^§^
12	F, 41	Gluteus		II	Surgical excision	–	–	Post-surgical evaluation
13	F, 70		Inguinal lymph node	III	Lymph node excisional biopsy	–	–	Staging
14^*^	M, 80		Femoral lymph node	III	Lymph node excisional biopsy	RT (72)	–	Re-staging^#^
15^*^	M, 74	Gluteus		IV	Surgical excision + loco-regional lymphadenectomy	RT (96), CHT (1)	–	Post-CHT evaluation

*MCC* Merkel cell carcinoma, *PET/CT* positron emission tomography/computed tomography, *M* male, *F* female, *CHT* chemotherapy, *RT* radiotherapy

*Patient with recurrent/relapsed MCC

^#^For clinical progression

^§^For suspected recurrence at imaging

### Patient-based analysis

Eight out of 15 patients were classified as having loco-regional or distant MCC lesions according to reference measures (histology and/or clinical-radiological follow-up), whereas in the remaining 7/15 cases no evidence of disease was observed (as reported in Table 2). Both studies identified at least one abnormal area of tracer uptake in all eight patients with MCC-related lesions, resulting in 100% sensitivity (95% CI 67.5–100%). Regarding the seven patients with no evidence of disease, six were correctly classified as true negative by ^18^F-FDG PET/CT (85.7% specificity; 95% CI 48.7–97.4%) and five by ^68^Ga-SRI (71.4% specificity; 95% CI 35.9–91.8%), with no significant difference in specificity (*p* = 0.59). Both ^18^F-FDG and ^68^Ga-somatostatin analogs (^68^Ga-DOTATATE) misclassified the same patient (#9) as false positive, showing abnormal uptake at the site of primary MCC removal, then attributed to cutaneous post-surgical inflammation. Discordant results between ^18^F-FDG (true negative) and ^68^Ga-SRI (false positive) PET/CT were observed in patient #10 (focal ^68^Ga-DOTATOC liver activity, not confirmed by the subsequent follow-up examinations). Regarding the four patients diagnosed as UPMCC, both PET/CT scans correctly classified one patient as negative (#13) and the other three cases as positives (#4, #8, and #14), but neither ^18^F-FDG nor ^68^Ga-SRI PET/CT were able to identify potential primary MCC sites.

**Table 2:** Results of PET/CT imaging (patient-based) and impact on patient’s management

Patient	Pre-PET/CT morphological imaging		^18^F-FDG PET/CT	^68^Ga-SRI PET/CT	Change in management	Patient management		Follow-up (months)	Outcome
	Type	Result (findings)	Results (findings)	Results (findings)		Expected	Undertaken		
1	CT	Positive (residual primary MCC, Lns)	TP (residual primary MCC, Lns, liver)	TP (primary MCC, Lns)	Yes	Local RT	CHT	9	Dead in PD
2^a^	CT	Positive (Lns)	TP (Lns)	TP (Lns)	No	CHT	CHT	8	Dead in PD
3	CT	Positive/suspicious (lung)	TN	TN	Yes	Individualized treatment	Wait and see	67	Alive, NED
4	CT	Positive (1 residual Ln)	TP (1 Ln)	TP (1 Ln)	No	Surgery + RT (lymph node)	Surgery + RT (lymph node)	34	Dead in PD
5	US	Negative	TN	TN	No	Adjuvant RT (loco-regional lymph nodes)	Adjuvant RT (loco-regional lymph nodes)	54	Alive, NED
6	CT	Positive/suspicious (Lns, liver)	TN	TN	Yes	CHT	Adjuvant RT (loco-regional lymph nodes)	52	Alive, NED
7	CT, MRI	Positive (residual primary MCC)	TP (residual primary MCC)	TP (primary MCC)	No	CHT	CHT	43	Alive, NED
8	CT	Positive (Lns)	TP (Lns)	TP (Lns)	Yes	CHT	SS analogs treatment	8	Dead in PD
9	CT	Negative	FP^#^ (skin)	FP^#^ (skin)	No	Adjuvant RT (primary site + loco-regional lymph nodes)	Adjuvant RT (primary site + loco-regional lymph nodes)	27	Alive, NED
10^*^	US	Negative	TN	FP^§^ (liver)	No	Wait and see	Wait and see	66	Dead (MCC-unrelated)
11^*^	US	Positive/suspicious (Lns)	TP (Lns, skin)	TP (Lns, skin)	No	CHT	CHT	21	Dead in PD
12	CT, US	Negative	TN	TN	No	Wait and see	Wait and see	122	Alive, NED
13	CT, US	Negative	TN	TN	No	Wait and see	Wait and see	118	Alive, NED
14^*^	US	Positive (Lns)	TP (Lns, thoracic wall, adrenals)	TP (Lns, thoracic wall, adrenals)	No	CHT	CHT	7	Dead in PD
15^*^	CT	Positive (Lns)	TP (Lns)	TP (Lns)	No	CHT	CHT	9	Dead in PD

*PET/CT* positron emission tomography/computed tomography, ^*18*^*F-FDG* ^18^F-fluoro-deoxy-glucose, ^*68*^*Ga-SRI* ^68^Ga-somatostatin receptor imaging, *CT* computed tomography, *MRI* magnetic resonance imaging, *US* ultrasonography, *MCC* Merkel cell carcinoma, *Lns* multiple lymph nodes, *Ln* lymph node, *TP* true positive, *TN* true negative, *FP* false positive, *RT* radiotherapy, *CHT* chemotherapy, *SS* somatostatin, *PD* progression disease, *NED* no evidence of disease

*Patient with recurrent/relapsed MCC

^#^Radiotracer uptake at the site of primary MCC removal, then attributed to cutaneous post-surgical inflammation

^§^Focus of liver activity not confirmed at following imaging

### Lesion-based analysis

**Fig. 1:**
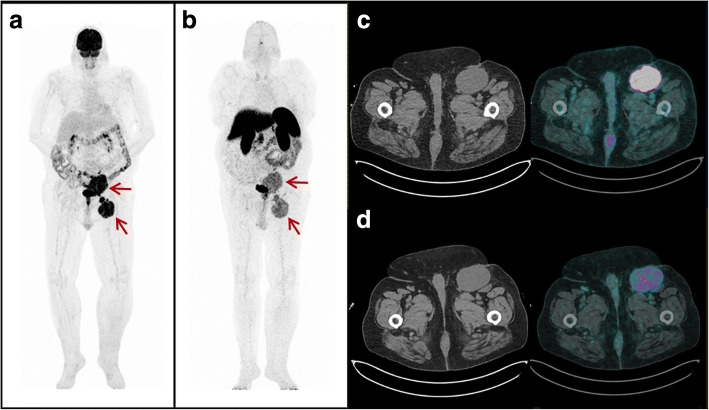
PET/CT images performed after chemotherapy in patient #8, who presented with UPMCC diagnosed by left inguinal lymph node biopsy. Maximum intensity projection (MIP) ^18^F-FDG PET/CT (**a**) and ^68^Ga-somatostatin analog (**b**) PET/CT images concordantly showed abnormal tracer uptake in multiple left iliac and inguinal lymph nodes (red arrows). Transaxial ^18^F-FDG (**c**), and ^68^Ga-somatostatin analog (**d**) PET/CT images concordantly showed abnormal tracer uptake (higher with ^18^F-FDG) in enlarged pathological left inguinal lymph node

A total of 75 foci of abnormal uptake using either ^18^F-FDG or ^68^Ga-SRI PET/CT in 8 patients were finally classified as MCC lesions. The reference standard was histology in 1/8 patients, morphological and/or functional imaging (diagnostic CT, MRI, PET/CT) in 5/8, and clinical plus morphological data (ultrasound imaging, CT) in 2/8. Sites of uptake were mainly represented by lymph nodes (*n* = 66), followed by skin (*n* = 4), liver (*n* = 2), adrenal glands (*n* = 2), and thoracic wall (*n* = 1). Two patients had only 1 lesion, 3 patients had less than 10 lesions, and 3 patients had 10 or more lesions. ^18^F-FDG PET/CT detected 67/75 of lesions (89.3%; mean SUVmax = 10.3 ± 6.9, range 1.9–48.9) and ^68^Ga-SRI PET/CT identified 69/75 of lesions (92%; mean SUVmax = 7.1 ± 3.7, range 1.9–19.9), with higher SUVmax at ^18^F-FDG than at ^68^Ga-SRI PET/CT (*p* = 0.001), whereas no significant differences either in the number of overall detected lesions (*p* = 0.72) or in the number of each specific type of lesion were found (see Table 3, Fig. 1). Overall, 61 lesions (81%) were identified by both tracers (54 lymph nodes, 4 skin lesions, 2 adrenal metastases, and 1 nodule of the thoracic wall), 6 lesions (8%) were detected only by ^18^F-FDG (4 pathological lymph nodes and 2 liver metastases), and 8 lesions (11%) were evident only with ^68^Ga-somatostatin analogs (all lymph node metastases).

**Table 3:** Results of PET/CT on a lesion-based analysis

Lesions (*n* = 75)	^18^F-FDG (detected lesions/total)	^68^Ga-somatostatin analogs (detected lesions/total)	*p* value
Lymph node	58/66	62/66	n.s.
Skin	4/4	4/4	n.s.
Liver	2/2	0/2	n.a.
Adrenal gland	2/2	2/2	n.s.
Thoracic wall	1/1	1/1	n.s.
Overall	67/75 (89.3%)	69/75 (92%)	n.s.
SUVmax (mean and SD)	10.3 ± 6.9	7.1 ± 3.7	0.001

*PET/CT* positron emission tomography/computed tomography, ^*18*^*F-FDG* ^18^F-fluoro-deoxy-glucose, *N.S.* not significant, *N.A.* not applicable, *SUVmax* maximum standardized uptake value, *SD* standard deviation

### Patient management

Additional information provided by ^18^F-FDG and/or ^68^Ga-SRI PET/CT influenced clinical decisions and led to a change in patient’s management in 4/15 patients (27%), as detailed in Table 2. In one patient (patient #1), the clinical strategy was influenced by specific findings identified by ^18^F-FDG alone (liver metastases which lead to an upstaging to stage IV), in another one (patient #8) by ^68^Ga-somatostatin analogs (due to the evidence of SSTR expression, he could be addressed to a palliative/second-line treatment with “cold” somatostatin analogs), while in the remaining two cases (patients #3 and #6) both tracers had the same clinical impact (not confirming radiologically suspected metastases).

## Discussion

To the best of our knowledge, this is the first original study directly comparing the diagnostic performance of ^18^F-FDG and ^68^Ga-SRI PET/CT in a series of patients with MCC. We found that ^18^F-FDG and ^68^Ga-somatostatin analogs provide good and equivalent diagnostic performance, with no significant differences either on a patient-based analysis or on a lesion-based analysis. From literature data, only few MCC patients have been imaged using both PET tracers, with better or equivalent performance of ^68^Ga-SRI compared to ^18^F-FDG PET/CT [25–27]; however, they are all case reports and, therefore, not reliably comparable with our findings. Similarly, our findings are not comparable with those reported by Lu et al. [33] in the largest series of nine MCC patients who were investigated comparing ^18^F-FDG PET/CT to ^111^In-pentetreotide SRS. In this series, ^18^F-FDG PET/CT performed better than SRS, but as suggested by the authors, this finding could be more related to the better spatial resolution and image quality of PET than to a real advantage of a metabolic tracer (glucose analog) over a receptor tracer (radiolabeled somatostatin analog). This hypothesis seems to be confirmed by our results. Indeed, in our series of patients investigated by PET/CT, ^18^F-FDG and ^68^Ga-somatostatin analogs provided comparable results in terms of sensitivity (100%) and specificity (86 and 71%, respectively). Also considering the overall lesion detection rate, no significant differences between the two tracers were found (89% for ^18^F-FDG vs 92% for ^68^Ga-somatostatin analogs), although ^68^Ga-SRI PET/CT failed to detect two liver lesions that were, on the contrary, detected by ^18^F-FDG leading to a change of both patient’s staging and therapy planning (patient #1). A possible explanation of this failure could be linked to the heterogeneous somatostatin receptor (SSTR) expression among MCC lesions, as observed by Gardair et al. [34]. These authors analyzed 105 tissue samples from 98 MCC patients by immunohistochemistry and demonstrated different degrees of expression and distribution of SSTR subtypes. Therefore, in our case (patient #1), we cannot exclude a heterogeneous SSTR expression in liver metastases (SSTR-PET negative) compared to the primary MCC lesion (SSTR-PET positive), although immunohistochemistry to confirm our hypothesis was not available.

**Fig. 2:**
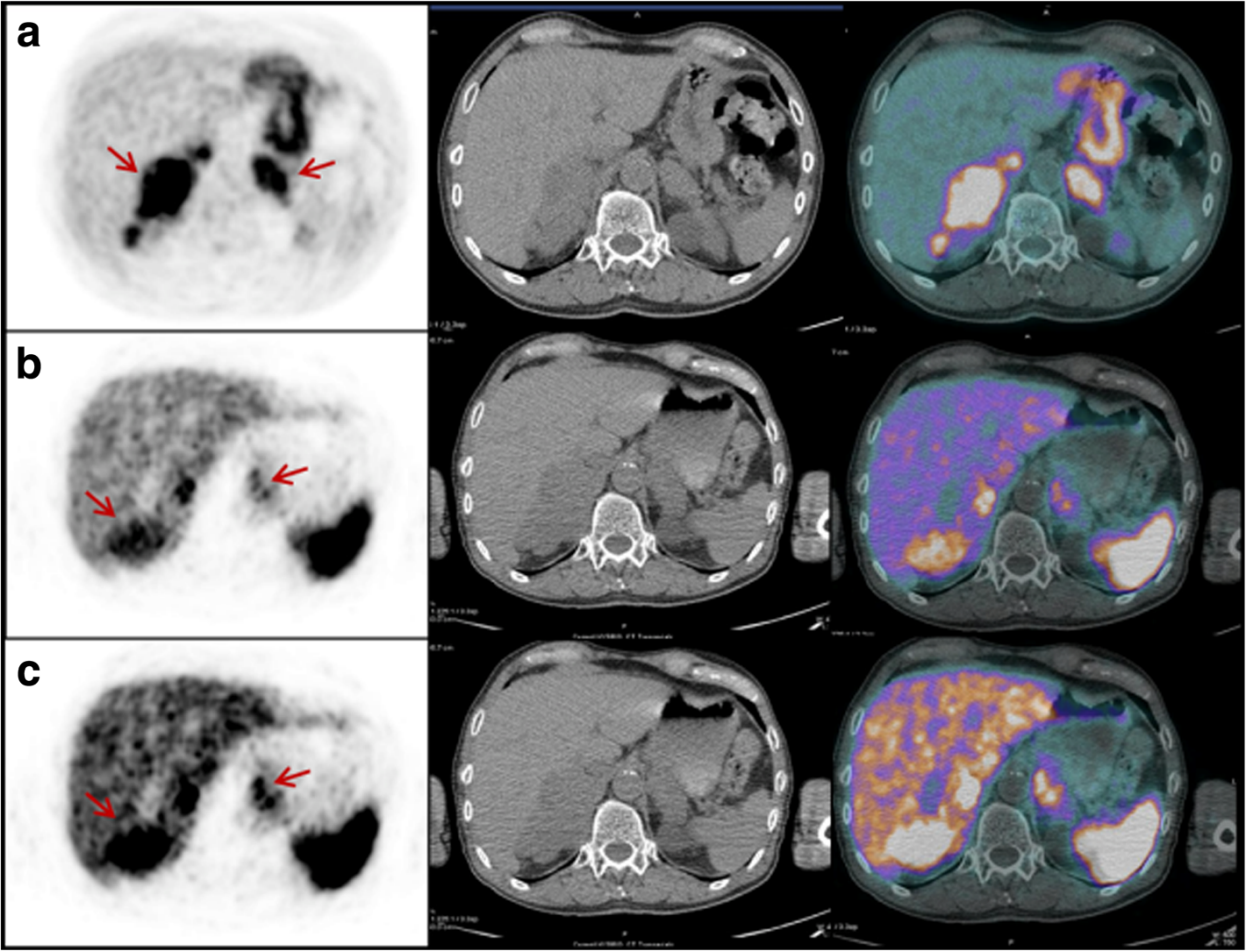
PET/CT images performed for re-staging in patient #14. Transaxial ^18^F-FDG (**a**) and ^68^Ga-somatostatin analogs (**b**, **c**) at different intensity levels. PET/CT images showed abnormal tracer uptake in both adrenal metastatic lesions (red arrows). Both adrenals were enlarged (right > left). The pattern of uptake was inhomogeneous with both ^18^F-FDG and ^68^Ga-somatostatin analogs and, in the whole, a “reverse” and complementary distribution of uptake was evident with the two tracers, with clearly parts of the tumor that take up one or the other tracer (e.g., the “hottest” part of the right adrenal at ^18^F-FDG appears substantially “cold” at ^68^Ga-SRI)

Notably, some organs may be not well explored with ^68^Ga-SRI since tracer is physiologically taken up by their cells. Malignant lesions have different patterns and intensities of ^68^Ga-somatostatin analog uptake compared to non-malignant tissues, also in sites characterized by high physiological background (e.g., the uncinated process of the pancreas and adrenals), as described by Kroiss et al. [35] in one of the largest cohort of NET patients imaged with ^68^Ga-DOTATOC. Accordingly, in our series, also adrenal metastases (patient #14) were easily identified at ^68^Ga-SRI PET/CT since the pattern of uptake was really inhomogeneous, its intensity was higher than liver, and the glands were enlarged (Fig. 2). In any case, the physiological tracer distribution should be always taken into account, with awareness that some metastases, especially small ones, could remain undiagnosed.

When comparing SUVmax values of the two tracers, we observed that the whole ^18^F-FDG uptake was higher than ^68^Ga-somatostatin analogs, suggesting that in our MCC population the tumor aggressiveness—as reflected by glucose avidity—seems to be a predominant characteristic. In other neuroendocrine tumors, particularly those of the gastro-entero-pancreatic tract, a positive ^68^Ga-SRI (representing the expression of SSTRs) with a faint or absent ^18^F-FDG uptake is typically observed in well-differentiated tumors with good prognosis, and a positive ^18^F-FDG with a faint or absent ^68^Ga-SRI uptake (representing the loss of SSTRs) is typically observed in more aggressive undifferentiated tumors [36, 37]. From our findings, MCC seems to deviate from this general “rule,” and shows a similar uptake pattern using both PET tracers. In support of these observations, Gardair et al. [34] argued that the expression of at least one SSTR in the majority of MCC lesions was an unexpected finding, as MCCs are poorly differentiated, highly proliferative tumors. Moreover, SSTR expression was not associated with clinical characteristics, Ki67 proliferative index, or clinical outcome, suggesting that the expression of SSTR is not itself a favorable prognostic index in MCC, differently from other neuroendocrine tumors. To explain this peculiar functional PET behavior of MCC, we may refer to one of the postulated theories on the controversial origin of MCC cell: a totipotent stem cell that acquires neuroendocrine characteristics (as SSTRs expression) during malignant transformation [38, 39].

In our series, UPMCC occurred in 4/15 patients (26%), localizing at the iliac-inguinal region in all cases. Our results are in accordance with the estimated incidence of UPMCC (as high as 25% of all MCC) and with the clinical site of presentation, being inguinal lymph node area one of the most common involved site at diagnosis [5, 40]. As previously mentioned, also in our series, both tracers could confirm lymph node involvement or detect unknown metastatic localizations, but were unable to identify the primary tumor [12]. Actually, in UPMCC, it has not yet been delineated if the tumor arises de novo from neural cells located within the involved lymph nodes or if the primary lesion undergoes spontaneous regression [5, 40].

We are aware that the retrospective nature of our study and the variable treatment protocols among different clinical institutions may lead to non-univocal results in terms of impact on patient management. However, we observed that additional information provided by ^18^F-FDG and/or ^68^Ga-SRI PET/CT influenced clinical decisions in 4/15 patients (27%). In particular, in one case (patient #1) ^18^F-FDG lead to a change of tumor staging (from III to IV) detecting two additional foci of increased uptake in the liver, not evident neither at ^68^Ga-DOTANOC nor at previous diagnostic CT and subsequently confirmed as metastatic lesions. With respect to this finding, although no statistically significant difference between the diagnostic performance of the two tracers was found, we retain that the overall performance of ^18^F-FDG and ^68^Ga-SRI PET/CT cannot be considered completely equal from a clinical perspective. Therefore, as shown by our data and reported in the NCCN guidelines [17], we retain that, in clinical practice, ^18^F-FDG PET/CT should not be replaced by ^68^Ga-SRI, which should be performed in addition, if clinically indicated. Anyway, it is important to consider that the decision on which tracer to employ at first line should be strongly influenced by the information that is considered more relevant for clinical management, in the perspective of “personalized medicine.” In this regard, ^18^F-FDG PET/CT may also add prognostic information, as recently suggested [11, 41], while ^68^Ga-SRI PET/CT may be useful for both therapy planning and response assessment in a “theranostic strategy” when peptide receptor radionuclide therapy (PRRT) is considered as a further potential treatment option [24, 25, 27, 35, 42]. Moreover, with the advent of immunotherapy with immune checkpoint inhibitors, PET/CT imaging with the two different tracers may turn out to play a role in a tailored-treatment approach providing useful and complementary information also in the setting of response assessment and follow-up for advanced MCC [43, 44].

Although our study represents to date the largest comparative series of MCC patients imaged by ^18^F-FDG and ^68^Ga-SRI PET/CT, it is affected by some limitations, mainly represented by its retrospective nature and the limited number of patients. However, the low incidence of MCC needs to be considered. Moreover, phenotypic tumor properties (i.e., proliferative index) and immunohistochemical characteristics were available only in few patients, preventing the correlation of tumor features and PET/CT results. Another potential limitation of this study concerns the use of three different ^68^Ga-SRI with different receptor affinities and pharmacokinetics. However, although some studies comparing different ^68^Ga-labeled peptides found the superiority of one radiopharmaceutical over the other, at present, the observed differences are not considered clinically relevant [45].

## Conclusions

^18^F-FDG and ^68^Ga-SRI PET/CT provide good and equivalent diagnostic performance in MCC patients, adding interesting insights into the complex biology of this rare tumor. However, these results do not suggest that ^18^F-FDG PET/CT should be replaced by ^68^Ga-SRI, which should be performed in addition, according to clinical indication, to the perspective of “personalized medicine.” Further data are recommended to assess the proper role of both PET tracers in MCC diagnostic imaging and patient management, particularly in patients undergoing immunotherapy.

## Abbreviations

^18^F-FDG: ^18^F-fluoro-deoxy-glucose
^68^Ga-SRI: ^68^Ga-somatostatin receptor imaging
MCC: Merkel cell carcinoma
SRS: Somatostatin receptor scintigraphy
UPMCC: MCC of unknown primary origin
